# POLR3B-Related Hypomyelinating Leukodystrophy Type 8 (4H Syndrome): A Case Series of Two Siblings

**DOI:** 10.7759/cureus.90642

**Published:** 2025-08-21

**Authors:** Daya Mani Jacob, Divyashri R Nagarajan, Sathya J Kakade

**Affiliations:** 1 Internal Medicine, Burjeel Medical City, Abu Dhabi, ARE; 2 Ophthalmology, Burjeel Medical City, Abu Dhabi, ARE

**Keywords:** 4h syndrome, consanguinity, developmental delay, hypomyelinating leukodystrophy, optic disc pallor, orthopedic intervention, polr3b mutation

## Abstract

We present a case series of two siblings from a consanguineous family with genetically confirmed POLR3B-related hypomyelinating leukodystrophy type 8 (4H syndrome), a rare autosomal recessive disorder characterized by hypomyelination, hypodontia, and hypogonadotropic hypogonadism. Despite sharing the same homozygous mutation, the siblings exhibited distinct clinical phenotypes, with the older child presenting with severe motor dysfunction, optic disc pallor, and requiring orthopedic surgery, while the younger maintained independent ambulation with milder neurological symptoms. Both exhibited high myopia and developmental delay, highlighting the multisystem nature of the disease.

## Introduction

Hypomyelinating leukodystrophies are a group of rare inherited disorders characterized by insufficient myelin formation in the central nervous system (CNS), resulting in progressive neurological dysfunction. Among them, 4H syndrome (hypomyelination, hypodontia, and hypogonadotropic hypogonadism) is a clinically and genetically heterogeneous disorder, primarily caused by mutations in POLR3A, POLR3B, and, more recently, POLR1C genes [1]. First described in 2011, 4H syndrome is classified under hypomyelinating leukodystrophy type 8 (HLD8) when associated with biallelic POLR3B mutations [2,3]. Although the exact prevalence remains unknown due to the rarity of the condition, POLR3-related leukodystrophies have emerged as one of the more commonly identified genetic causes of hypomyelination in children, particularly with the increasing use of next-generation sequencing [4,5].

The clinical phenotype of 4H syndrome includes early-onset developmental delay, ataxia, dysarthria, tremors, visual impairment, dental abnormalities, and pubertal delay. MRI typically reveals diffuse cerebral hypomyelination. A multidisciplinary approach is crucial for managing the neurological, orthopedic, endocrine, ophthalmological, and developmental aspects of the disorder, as there is currently no curative treatment or disease-modifying therapy available [6].

This case series describes two siblings from a consanguineous family with genetically confirmed POLR3B-related leukodystrophy, highlighting intrafamilial phenotypic variability and underscoring the need for coordinated multidisciplinary management.

## Case presentation

Case 1

A 13-year-old male, known to have a homozygous POLR3B (c.2930T>A; p.Val977Asp) gene mutation confirmed by genetic testing, was referred from the neurology clinic for an eye check-up due to defective vision that had been ongoing for 30 days. He has a confirmed diagnosis of leukodystrophy, hypomyelination type 8, also referred to as 4H syndrome (hypomyelination with hypogonadotropic hypogonadism and hypodontia), which was established at the age of 11 years following evaluation for progressive neurological symptoms and confirmatory genetic analysis.

His symptoms include global developmental delay, progressive gait disturbance, dystonia, dysarthria, dysphagia, speech delay, poor appetite, tremors, weakness, Achilles tendinitis, right leg Achilles tendon contracture, acquired deformity of the right lower leg, acquired pes planovalgus of the right foot, and carnitine and vitamin D deficiencies. He underwent surgical correction for right gastrocnemius recession and pes planovalgus deformity.

He also has a history of failure to thrive (BMI: 12.1 kg/m²), with a weight of 22 kg and a height of 135 cm, corresponding to the 0.14th percentile. Birth history indicates a full-term delivery at 38 weeks and six days, with a birth weight of 3.495 kg and no reported neonatal complications. His medications include oral arginine 1 ampoule daily, oral cholecalciferol 25,000 IU weekly, and oral levocarnitine 1000 mg twice daily. 

MRI of the brain done at another facility showed hypomyelination, and genetic testing confirmed the diagnosis of hypomyelinating leukodystrophy type 8 with or without oligodontia and/or hypogonadotropic hypogonadism. 

**Figure 1:**
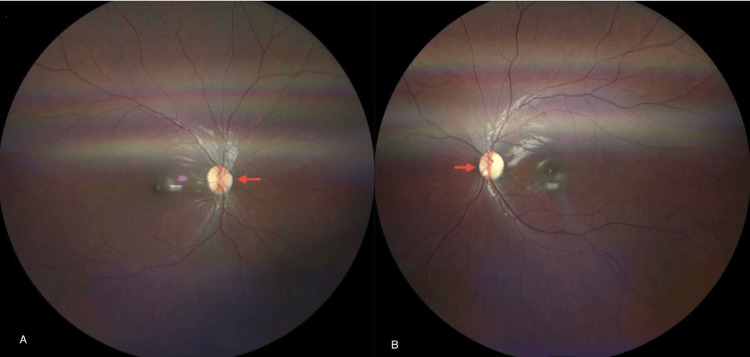
Fundoscopy A) Right eye showing optic disc pallor. B) Left eye showing optic disc pallor. Optic disc pallor in 4H syndrome reflects underlying optic nerve involvement secondary to hypomyelination, contributing to progressive visual impairment.

As part of his systemic assessment, the patient underwent a comprehensive ophthalmologic examination. Refraction testing revealed high myopia with astigmatism in both eyes, detailed in Table 1. Fundoscopic examination revealed bilateral optic disc pallor, suggesting underlying optic nerve involvement (Figure 1). 

**Table 1:** Refractive error on ophthalmologic examination

Eye	Spherical refraction	Cylindrical refraction
Right (OD)	-8.50	-1.25
Left (OS)	-10.25	-1.00

He is currently ambulant with support; however, his left foot has begun to evert. He continues to receive physiotherapy and speech therapy and has been reviewed by the rehabilitation team. The family history is significant for parental consanguinity, and two siblings are affected with the same disease. A maternal cousin is reported to have similar symptoms, including tremors and low IQ, and is being evaluated for a possible white matter disorder. He lives at home with his parents and siblings, with no special home equipment in use.

Case 2 

An 11-year-old male with a confirmed POLR3B gene mutation associated with hypomyelinating leukodystrophy type 8 (4H syndrome) was evaluated for progressive lower limb deformities. He was born at term (38 weeks and six days of gestation) via an unremarkable delivery, with a birth weight of 3.495 kg and no neonatal complications. Developmental delays were noted early in life, and subsequent evaluation by the neurology and genetics departments at another facility confirmed the diagnosis. Genetic counseling was provided to the family.

His medical history is significant for developmental delay, dystonia, and gait imbalance, but there is no reported history of dysphagia. He has no history of previous surgeries or chronic medication use. The family history is notable for parental consanguinity, a similarly affected sibling, and a maternal cousin with tremors and low IQ, suspected to have a white matter disorder.

The patient attends a special education school and is followed regularly by pediatric neurology and orthopedic rehabilitation services. In preparation for bilateral lower limb corrective surgery, he was evaluated in both the orthopedic and pediatric cardiology clinics. Clinically, he was found to ambulate independently but had bilateral pes planovalgus deformity, which was correctable with tiptoeing, as well as tightness of the gastrocnemius complex bilaterally.

On physical examination, he had a normal ankle range of motion (neutral to 30° plantarflexion), intact ankle ligaments, mobile subtalar joints, flat feet when standing, and no limb length discrepancy. He underwent bilateral subtalar arthroereisis, bilateral gastrocnemius lengthening, and bilateral guided growth of the medial distal tibia.

**Figure 2:**
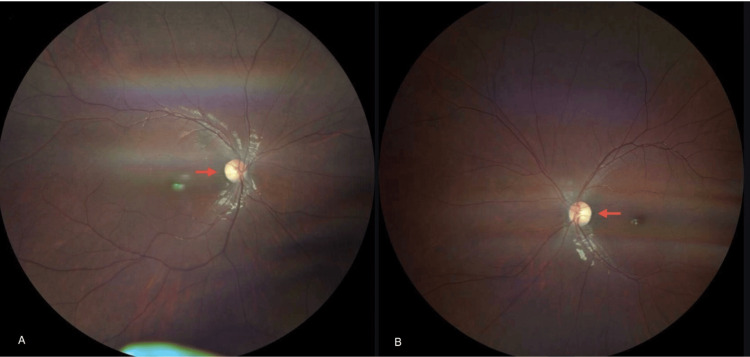
Fundoscopy A) Right eye showing pale optic disc suggestive of optic atrophy with normal retinal vasculature and macula. B) Left eye showing pale optic disc suggestive of optic atrophy with normal retinal vasculature and macula.

The patient underwent an ophthalmic examination as part of his multisystem assessment. Refraction testing revealed bilateral high myopia with astigmatism, as summarized in Table 2. Fundoscopy was also performed (Figure 2).

**Table 2:** Refractive error on ophthalmic examination

Eye	Spherical refraction	Cylindrical refraction
Right (OD)	-6.00	-1.50
Left (OS)	-5.75	-1.25

Following surgery, he began mobilizing with one-person assistance. He currently participates in physiotherapy to support mobility, speech therapy for communication and swallowing, occupational therapy for fine motor skills and cognitive support, and hydrotherapy to improve gait and manage tremors.

## Discussion

This case series focuses on the clinical spectrum and intrafamilial variability of POLR3B-related leukodystrophy (4H syndrome). Despite carrying the same homozygous mutation, the two siblings exhibited differing degrees of neurological impairment, functional status, and organ involvement. The older sibling demonstrated significant gait disturbance, speech impairment, and visual changes, while the younger had milder motor dysfunction and preserved ambulation. The differences in siblings with respect to presentation, interventions, and outcomes are summarized in Table 3.

**Table 3:** Comparison of clinical features, interventions, and outcomes in two siblings with POLR3B-related hypomyelinating leukodystrophy (4H syndrome)

Feature	Case 1 (13-year-old male)	Case 2 (11-year-old male)
Genetics	Homozygous POLR3B mutation	Homozygous POLR3B mutation
Birth history	Full-term, birth weight >3 kg, no neonatal complications	Full-term, birth weight 3.495 kg, no neonatal complications
Developmental course	Global developmental delay, speech delay	Global developmental delay, attends special education school
Neurological features	Gait disturbance, dystonia, tremors, weakness, dysarthria, dysphagia	Gait imbalance, dystonia, no dysphagia
Musculoskeletal findings	Achilles tendinitis, right Achilles tendon contracture, right pes planovalgus deformity	Bilateral pes planovalgus (correctable), bilateral gastrocnemius tightness
Nutritional status	Failure to thrive (BMI 12.1, weight 22 kg, height 135 cm, <1st percentile), poor appetite	No reported severe FTT; weight/height not detailed
Ophthalmologic findings	High myopia with astigmatism (OD: -8.50/-1.25, OS: -10.25/-1.00), optic disc pallor	High myopia with astigmatism (OD: -6.00/-1.50, OS: -5.75/-1.25), optic disc atrophy
Other systemic findings	Vitamin D deficiency, carnitine deficiency	No metabolic deficiencies reported
Interventions (surgical)	Right gastrocnemius recession, right pes planovalgus correction	Bilateral subtalar arthroereisis, bilateral gastrocnemius lengthening, bilateral guided growth of medial distal tibia
Interventions (medical/rehabilitative)	Oral arginine, levocarnitine, cholecalciferol; physiotherapy; speech therapy	Post-surgical rehab with physiotherapy, speech therapy, occupational therapy, hydrotherapy; no chronic medications
Functional status	Ambulates with support; left foot beginning to evert	Ambulates independently pre-surgery; requires one-person assist post-surgery; improving with therapy
Family history	Parental consanguinity, two siblings affected, maternal cousin with tremors and low IQ	Parental consanguinity, two siblings affected, maternal cousin with tremors and low IQ
Outcome (current)	Ambulatory with support, progressive musculoskeletal issues, continues therapy	Mobilizing with assistance post-surgery, receiving multidisciplinary rehabilitation

Pathogenic variants in POLR3B are among the most frequently reported causes of 4H syndrome. The most common recurrent mutation is c.1568T>A (p.Val523Glu), often seen in compound heterozygous or homozygous states, accounting for a substantial proportion of cases worldwide [7]. Other reported variants include missense, nonsense, frameshift, and splice-site mutations distributed across the gene. Current evidence suggests that while most POLR3B mutations converge on a similar phenotype of hypomyelination with variable neurologic and endocrine features, some genotype-phenotype correlations have been proposed. For example, individuals with homozygous missense variants often present with a milder disease course and later onset, whereas those with truncating or splice-site variants may develop earlier and more severe motor and cognitive impairment [6].

Optic disc pallor, which is a sign of optic nerve atrophy, has also been documented in patients with POLR3-related leukodystrophies and correlates with CNS hypomyelination [6]. Bettinger et al. (2024) reported that 68% of patients with leukodystrophies exhibited neuro-ophthalmologic symptoms, including optic atrophy, visual neglect, strabismus, and nystagmus [8]. This further supports the presence of optic atrophy in 4H leukodystrophy, which is part of the POLR3-related leukodystrophy spectrum characterized by widespread CNS hypomyelination, including the optic nerves.

The occurrence of these symptoms was early or late based on the subtype of leukodystrophy, which accentuated the differences in how the visual pathways are affected in these disorders.

Musculoskeletal abnormalities such as pes planovalgus and tendon contractures are also well-recognized complications. These may require surgical intervention, as illustrated in Case 2, where orthopedic correction combined with physiotherapy and gait training significantly improved mobility and quality of life [9]. Neurologic care for all leukodystrophies must be tailored to the patient's stage of the disease (presymptomatic, early, intermediate, or advanced), taking into consideration particular symptoms and existing knowledge gaps [10]. Endocrine involvement in 4H syndrome commonly presents as hypogonadotropic hypogonadism, leading to delayed puberty. This results from impaired gonadotropin-releasing hormone (GnRH) secretion, receptor dysfunction, or reduced bioactivity of GnRH. Affected individuals typically exhibit abnormal baseline FSH and LH levels, with a blunted or absent response to GnRH stimulation testing [11].

Given the autosomal recessive inheritance pattern, consanguinity significantly increases the risk of homozygous pathogenic variants. In this context, genetic counseling was essential to address reproductive risks and initiate cascade testing in extended family members [12].

Emerging therapeutic strategies are being investigated to move beyond supportive management. Metabolic support with the ketogenic diet and regulation of iron metabolism have shown promise in promoting myelin repair by enhancing brain energy supply and correcting metabolic dysfunction in oligodendrocytes [13]. Additionally, recent translational studies suggest that gene-targeted approaches, including antisense oligonucleotides and adeno-associated viral (AAV)-mediated gene therapy, may hold potential for POLR3-related disorders, though they remain in preclinical stages [14]. Remyelination-promoting compounds, such as clemastine fumarate, have also demonstrated efficacy in enhancing oligodendrocyte differentiation in leukodystrophy models and are being explored as potential adjuncts [15]. These strategies highlight the shift from purely symptomatic treatment toward disease-modifying interventions, though clinical application in 4H syndrome remains investigational.

In children with 4H syndrome, visual rehabilitation focuses on improving daily functioning and learning through regular eye check-ups, use of low vision aids such as magnifiers or screen readers, eye movement exercises for problems with focusing or tracking, and support at school with tools like large print materials, extra time for tasks, and multisensory learning methods to help them keep up with their education despite progressive vision loss [16]. 

Overall, effective management of 4H syndrome requires a coordinated multidisciplinary approach involving neurology, rehabilitation, orthopedics, ophthalmology, endocrinology, and genetics. Supportive therapies, including speech, physical, occupational, and nutritional interventions, are critical to preserving function. Regular surveillance of endocrine function and pubertal progression remains essential, as hormonal deficiencies may become apparent later in the disease course.

## Conclusions

This family cluster highlights the variable expression of 4H syndrome due to POLR3B mutations, the importance of early diagnosis, and the value of comprehensive, multidisciplinary care. Recognition of key clinical features such as developmental delay, gait abnormalities, and optic disc pallor should prompt genetic evaluation. Individualized therapy, including surgical correction when necessary, can substantially impact functional outcomes and quality of life.
